# Broken Scalpel Blade During Emergent Cricothyroidotomy: An Unexpected Complication in a Critical Situation

**DOI:** 10.7759/cureus.8868

**Published:** 2020-06-27

**Authors:** Arindam Sharma, Shreyak Sharma, Arunima Sharma, Khawaja Muddassir

**Affiliations:** 1 Internal Medicine, University of Tennessee Health Science Center, Memphis, USA; 2 Renal Medicine, Brigham and Women's Hospital, Boston, USA; 3 Internal Medicine, Sikkim Manipal Institute of Medical Sciences, Gangtok, IND; 4 Pulmonary and Critical Care, University of Tennessee Health Science Center, Memphis, USA

**Keywords:** cricothyroidotomy, emergency, airway access, head and neck surgery

## Abstract

Scalpel-bougie cricothyroidotomy is the most common surgical procedure to obtain emergency airway access when routine methods fail. We present a case of a broken scalpel blade during emergency cricothyroidotomy further complicating respiratory access.

## Introduction

A difficult airway is defined as a clinical situation in which a conventionally trained anesthesiologist experiences difficulty with facemask ventilation of the upper airway, difficulty with tracheal intubation, or both [1]. A ‘Can’t Intubate Can’t Oxygenate’ situation is one of the most challenging events in difficult airway management. This occurs when attempts to manage a patient’s airway with tracheal intubation, face mask ventilation, or placement of a supraglottic airway device have failed. Airway management in such situations is time sensitive as a delay poses a significant risk of hypoxic brain damage and death [2]. The scalpel-bougie cricothyroidotomy technique is the most reliable method of obtaining emergency front-of-neck access to the trachea in such situations [3,4]. However, unexpected complications can occur during this emergent procedure as well. We present a case where emergent surgical cricothyroidotomy was complicated by breaking of the scalpel blade and its entrapment in the soft tissue of the neck.

## Case presentation

A 68-year-old female presented to the emergency department with breathing difficulty, swelling of throat and tongue, and rashes all over the body. The symptoms had started within minutes of turning on the air conditioner in a hotel room.

She was allergic to penicillin and erythromycin. She did not have food allergies or a previous history of anaphylaxis. Her only medication was levothyroxine. Upon arrival, she was tachypneic with a respiratory rate in the 20s with oxygen saturation 96% on 3L oxygen by nasal cannula. Examination showed significant edema of the soft palate, posterior oropharynx, and uvula. She had difficulty with tongue protrusion. Her neck was large, and palpation of laryngeal landmarks was difficult. She exhibited a weak muffled voice and had mildly labored respirations.

Nasotracheal intubation via flexible endoscope was attempted. There was difficulty in passing the endoscope due to the patient's swollen nasopharynx and oropharynx, causing difficulty in visualization of the supraglottic larynx. Once this was attained, significant supraglottic edema was observed with difficulty in visualizing vocal cords. Upon passing the scope through the vocal cords, the patient developed laryngospasm. An attempt was made to ventilate the patient with a bag and mask, which was unsuccessful. Attempt to view the supraglottic larynx with the C-MAC video (Karl Storz, Tuttlingen, Germany) laryngoscope was also unsuccessful. 

**Figure 1:**
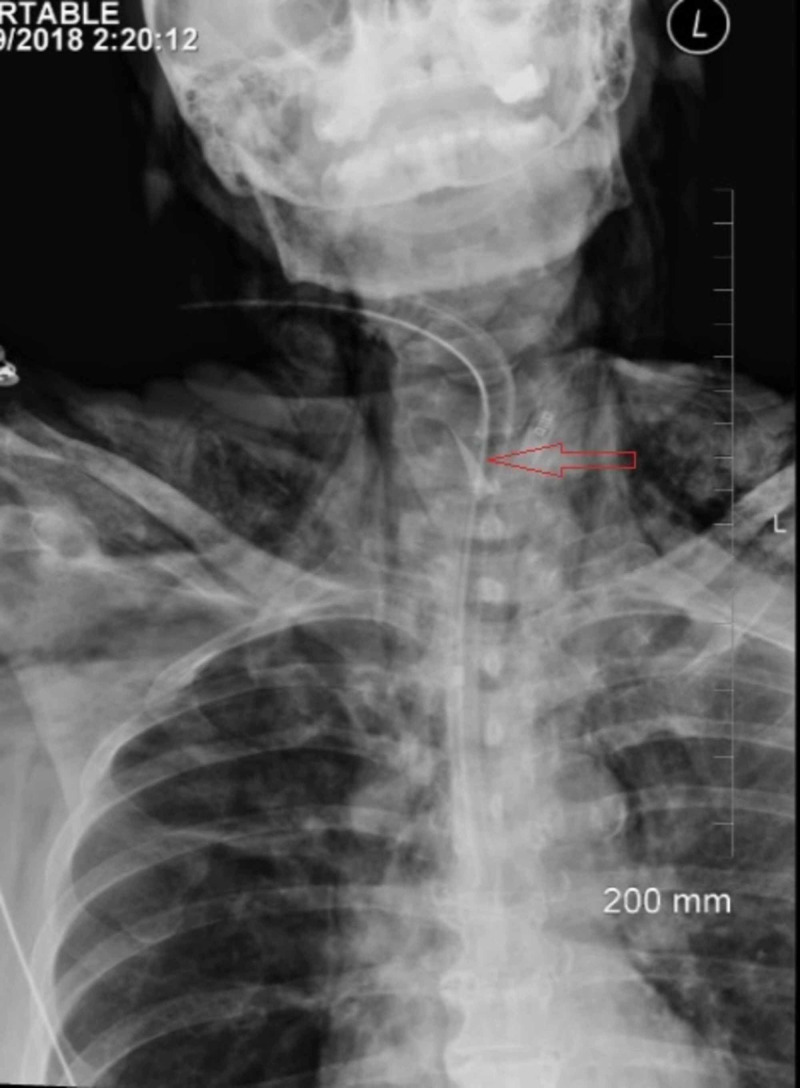
Chest X-ray anteroposterior view showing scalpel blade (labeled)

**Figure 2:**
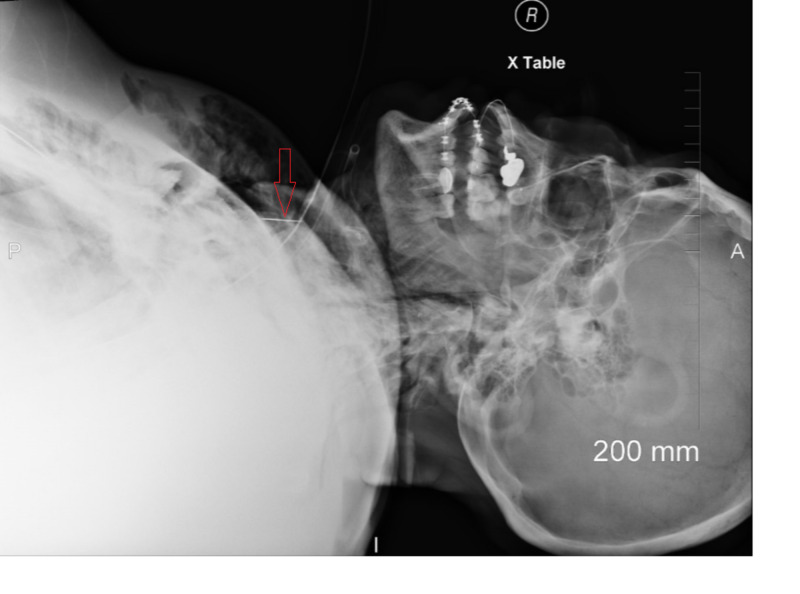
Chest X-ray lateral view showing scalpel blade (labeled)

The patient's oxygen saturation worsened, and a surgical airway was attempted. A vertical incision was made in the skin which was deepened with a scalpel. The thyroid cartilage was palpated and there was difficulty in palpating the cricoid cartilage. A surgical cricothyroidotomy was performed using a blade 11 scalpel and a bougie. However, the tip of the scalpel broke during the procedure and got lodged in the soft tissue of the neck (Figures 1-2). The endotracheal tube was successfully passed through the trachea, and the patient was taken to the operating room for removal of the scalpel tip. The scalpel tip was successfully removed, and the patient’s tracheostomy was formalized. The patient subsequently had an uneventful hospital course and the tracheostomy tube was removed after four days with the tracheotomy site left open for healing with secondary intention.

## Discussion

Emergent surgical cricothyroidotomy is a lifesaving intervention which is recommended as the final step in most difficult airway management algorithms [5].

This technique requires three standard pieces of equipment: (1) scalpel with size 10 blade (2) bougie with coude (angled) tip, and (3) a size 6 cuffed endotracheal tube. A transverse stab incision through the cricothyroid membrane is made which is then turned 90 degrees. The coude tip of the bougie is then slid along the blade into the trachea, followed by railroading of the cuffed endotracheal tube into the trachea. The tube is finally secured after ventilation, cuff inflation and confirmation of position with capnography. For individuals with an impalpable cricothyroid membrane, an 8-10 cm vertical incision over the larynx is made, caudad to cephalad. Blunt dissection is then used to identify the larynx and cricothyroid membrane. The remaining steps remain the same [3,6]. This technique has replaced needle or cannula cricothyroidotomy as the preferred technique for emergent front of the neck access [4,7,8].

A report from the United Kingdom [9] revealed that most routine tracheostomies are now performed percutaneously, and experience in surgical cricothyroidotomy is largely limited to senior Ear, Nose and Throat (ENT) surgeons and Oral and Maxillofacial surgeons. Many emergency personnel have limited exposure to this procedure, which may affect confidence and competence in performing this procedure in a high-pressure situation [6]. Therefore, proper training for this procedure using all available teaching methods including simulation is important for all professionals who may encounter difficult airway situations, such as emergency personnel, anesthesiologists and ENT surgeons.

Like any other procedure, surgical cricothyroidotomy can be complicated by unexpected events. Known early complications include injury to cartilaginous structures, failure to obtain airway, hemorrhage, prolonged execution time, pneumothorax and subcutaneous emphysema [10]. To our knowledge, this is the first reported case of a surgical blade breaking and getting lodged in the neck during surgical cricothyroidotomy. There have been a few case reports of scalpel blade breaking in other procedures, such as intervertebral disk surgery and hip and knee surgeries [11-15]. The mechanical properties of surgical instruments are designed based on the expected firmness of the tissues on which they would be used. Furthermore, limited area of manipulation in closed body spaces predisposes to bending of the surgical blade beyond its maximum bending stress resulting in breakage. This is the likely reason for most reported breakages of surgical blades involving hard tissues and in areas with room for mobility, such as arthroscopic joint surgery and intervertebral disk surgery.

In the present case, the neck was large and there was significant soft tissue swelling. The field of view in surgical cricothyroidotomy is limited, which can predispose to excessive bending of the scalpel blade leading to breakage. Care must be taken to avoid excessive bending of the scalpel blade in such scenarios. Defective equipment is also possible in this case and cannot be ruled out.

The breaking of the scalpel blade, in this case, did not interfere with the insertion of the bougie and endotracheal tube. However, breaking of the scalpel blade can complicate airway access if the blade gets lodged in the trachea and obstructs the airway. Entrapment of the blade in the distal airway can also predispose to infection and necessitate bronchoscopy or surgery for removal.

This case is a vivid educational example of the challenging nature of emergent airway access procedures and unexpected events that may be encountered in such situations. We hope this case will encourage readers to learn more about emergent airway access and increase efforts to obtain training in this procedure. This case also demonstrates the importance of thorough and periodic checking of equipment in emergency airway management kits. Equipment malfunction should be anticipated in emergency airway situations and spare equipment should always be readily available.

## Conclusions

A ‘Can’t Intubate Can’t Oxygenate’ situation is a critical airway management situation which requires a rapid and coordinated response to prevent patient harm. The scalpel-bougie technique is the mainstay for managing such situations. However, this technique can be complicated by breaking of the scalpel blade during the procedure. Equipment malfunction should be anticipated in all critical procedures and backup equipment should readily available.

## References

[1] Apfelbaum JL, Hagberg CA, Caplan RA (2013). Practice guidelines for management of the difficult airway: an updated report by the American Society of Anesthesiologists Task Force on management of the difficult airway. Anesthesiology.

[2] Joffe AM, Aziz MF, Posner KL, Duggan LV, Mincer SL, Domino KB (2019). Management of difficult tracheal intubation: a closed claims analysis. Anesthesiology.

[3] Frerk C, Mitchell VS, McNarry AF (2015). Difficult Airway Society 2015 guidelines for management of unanticipated difficult intubation in adults. Br J Anaesth.

[4] Hubble MW, Wilfong DA, Brown LH, Hertelendy A, Benner RW (2010). A meta-analysis of prehospital airway control techniques part II: alternative airway devices and cricothyrotomy success rates. Prehosp Emerg Care.

[5] Edelman DA, Perkins EJ, Brewster DJ (2019). Difficult airway management algorithms: a directed review. Anaesthesia.

[6] McNiven ND, Pracy JP, McGrath BA, Robson AK (2018). The role of scalpel-bougie cricothyroidotomy in managing emergency front of neck airway access. a review and technical update for ENT surgeons. Clin Otolaryngol.

[7] Pracy JP, Brennan L, Cook TM, Hartle AJ, Marks RJ, McGrath BA, Patel AN (2016). Surgical intervention during a Can't intubate Can't Oxygenate (CICO) event: emergency front-of-neck airway (FONA)?. Br J Anaesth.

[8] Lockey D, Crewdson K, Weaver A, Davies G (2014). Observational study of the success rates of intubation and failed intubation airway rescue techniques in 7256 attempted intubations of trauma patients by pre-hospital physicians. Br J Anaesth.

[9] (2020). Tracheostomy care: on the right trach?. https://www.ncepod.org.uk/2014tc.html.

[10] DeVore EK, Redmann A, Howell R, Khosla S (2019). Best practices for emergency surgical airway: a systematic review. Laryngoscope Investig Otolaryngol.

[11] Aras AB, Ozkan Of, Alar T (2014). A wandering intravascular scalpel fragment after lumbar discectomy: a case report. Turk Neurosurg.

[12] Zheng GB, Wang Z (2020). Removal of the deeply located intradiskal broken knife blade with arthroscopic assistance: case report and literature review. World Neurosurg.

[13] Agrawal Y, Sharma S, Chopra S, Purohit DK (2017). Retrieval of a retained broken scalpel blade from lumbar intervertebral disc space - a case report. Romanian Neurosurgery.

[14] Prasad R, Amstutz HC, Sparling EA (2000). Use of a magnet to retrieve a broken scalpel blade. J Arthroplasty.

[15] Gruson KI, Ilalov K, Youm T (2008). A broken scalpel blade tip: an unusual complication of knee arthroscopy. Bull NYU Hosp Jt Dis.

